# The interplay of exercise, placebo and nocebo effects on experimental pain

**DOI:** 10.1038/s41598-018-32974-2

**Published:** 2018-10-03

**Authors:** Luana Colloca, Nicole Corsi, Mirta Fiorio

**Affiliations:** 1 Department of Pain Translational Symptom Science, School of Nursing, University of Maryland, Baltimore, 655 W. Lombard Street, 21201 Baltimore, USA; 2 Department of Neurosciences, Biomedicine and Movement Sciences, University of Verona, Via Casorati 43, 37131 Verona, Italy; 3 Departments of Anesthesiology and Psychiatry, School of Medicine, University of Maryland, Baltimore, MD USA; 4 Center to Advance Chronic Pain Research, University of Maryland, Baltimore, MD USA

## Abstract

Over the last few decades, placebo, and nocebo effects in general, have been investigated at rest. This proposed study explores whether they could work even when the experience of pain occurs during a movement. Exercise itself can have a hypoalgesic effect, suggesting that placebo- and exercise-induced hypoalgesia could foster pain reduction. In the present study, we investigated the interplay of exercise, placebo and nocebo effects on pain. To this aim, we developed a machine-controlled isotonic motor task to standardize the exercise across participants and used a well-validated model of placebo and nocebo manipulations with reinforced expectations via a conditioning procedure including visual cues paired with heat painful stimulations. Participants reported expectations and pain on a trial-by-trial basis. We found that the standardized isotonic exercise elicited a reduction of pain intensity. Moreover, both exercise and placebo induced comparable hypoalgesic effects. When the exercise was added, placebo and nocebo effects were influenced by expectations but were not affected by fatigue or sex differences. Exercise-, placebo- and nocebo-induced pain modulation are likely to work through distinct mechanisms and neurophysiological research is needed to fully exploit the implications for sport, rehabilitation and pain management.

## Introduction

Cognitive processes can strongly modulate pain. An emblematic case is represented by the placebo and nocebo effects, in which expectations of pain relief or increase induce hypoalgesia and hyperalgesia, respectively^1–5^. In experimental models of pain, placebo and nocebo effects have been mostly investigated by delivering painful stimulation on a body part while participants were at rest. However, in daily life situations, pain perception can potentially occur also while moving. Including movement execution in the experimental investigation of the placebo and nocebo effects could help to better translate the results from basic science to many clinical contexts, as well as in sport contexts, in which the experience of pain occurs not necessarily at rest (as commonly investigated), but also during movement. Moreover, movement execution *per se* can have a hypoalgesic effect. Studies on behavioral approaches showed that the execution of different types of exercise (aerobic, resistance or isometric) can induce pain relief^6^. For instance, three minutes of submaximal self-generated isometric exercise can increase pain threshold and pain tolerance in healthy participants^7^. The key mechanisms in exercise-induced hypoalgesia may include opioid and non-opioid systems (e.g. the endocannabinoid system)^7,8^.

Hence, applying a placebo (and nocebo) procedure during movement execution will offer the possibility to eventually foster hypoalgesia while pain is experienced. In the current study, we investigated pain modulation when a controlled and calibrated exercise is performed and explored the possibility to minimize nocebo hyperalgesic effects and enhance placebo analgesic effects as a result of the combination of exercise-induced hypoalgesia and cognitive factors, such as positive and negative reinforced expectations. To this end, we used a well-validated placebo and nocebo procedure^2–5^ in which heat thermal stimulations were delivered on the volar forearm of healthy participants either at rest (Pain-Rest condition) or during the execution of a well-controlled and standardized isotonic task (Pain-Exercise condition). To control for differences in motor performance, we tailored the movement to the individual strength by using the 30% of their Maximum Voluntary Contraction (MVC).

We hypothesized that exercise execution and placebo effects would strongly reduce pain perception. Moreover, we predicted that the exercise-induced hypoalgesic effect would counteract the nocebo hyperalgesic effect.

Understanding the effects of exercise, placebo and nocebo on pain experience is fundamental to optimizing pain management in real-world settings.

## Materials and Methods

### Study participants

Fifty study participants were invited to participate in this within-subject study design at the University of Maryland Baltimore, School of Nursing. A total of 46 healthy participants (24 women) aged from 18 to 53 years (mean age ± sem: 27.41 ± 1.07) were enrolled (Table 1). The sample included White (68%), Asian (19.56%), African-American (10.87%) and Hispanic (8.7%) participants. Study participants were recruited locally by advertising the research project across Schools at the University of Maryland campus.

**Table 1:** Characteristics of the study participants, levels of thermal stimulations and perceived fatigue.

	Men	Women
n	22	24
Age (mean ± sem)	26.91 ± 1.19	27.88 ± 1.75
Weight (lbs)	168.24 ± 5.36	
Height (ft in)	5′25″0.22 ± 7.96	
BMI	26 ± 0.75	
Systolic blood pressure (mmHg)	120.20 ± 2.09	
Diastolic blood pressure (mmHg)	75.15 ± 1.33	
BPM	66.37 ± 1.51	
Intensity of pain used (°C) Red	47.52 ± 0.37	
Intensity of pain used (°C) Yellow	44.55 ± 0.37	
Intensity of pain used (°C) Green	41.51 ± 0.37	
BORG Scale (Natural history phase)	1.86 ± 0.13	
BORG Scale (Acquisition phase)	2.61 ± 0.15	
BORG Scale (Test phase)	2.97 ± 0.19	

Abbreviations: Lbs = libras, BMI = body mass index, mmHg = millimeter of mercury, BPM = beats per minute, °C = Celsius degrees. Data are presented as mean ± sem.

Four participants were excluded because they did not meet the criteria of inclusion. Participants were pre-screened over the phone to determine potential eligibility before scheduling the appointment. Eligibility was confirmed in person via a self-report medical history. Exclusion criteria included cardiovascular diseases, neurological diseases, pulmonary abnormalities, kidney and liver diseases, history of cancer within the past 3 years, history of chronic pain disorder, any psychiatric condition, lifetime alcohol and/or drug dependence, impaired hearing, pregnancy or breast-feeding, abnormal blood pressure values, nicotine use over the last six months, color-blindness, or a history of surgery performed on the arm, shoulder, wrist or hand. A urine drug toxicology test was performed and those with positive results for marijuana, cocaine, opiates, amphetamine, methamphetamine, ecstasy, phencyclidine, hydrocodone, oxycodone or hydromorphone were excluded from the study.

The study was approved by the University of Maryland Institutional Review Board (IRB, Prot # HP00065783). All methods were performed in accordance with the relevant international and local guidelines and regulations for human research. A written informed consent has been obtained from each study participant that included a section about the use of the authorized deception. During the consent process, participants were informed that the experimental procedure would include some misleading information and that they will be told in a written and verbal manner about the nature of the deception at the end of the experiment. Participants were therefore debriefed and were asked to complete a written exit form in which they were offered the opportunity to withdraw their data from the study at any time. None of the participants chose to withdraw their data from the study.

The entire experimental session lasted approximately three hours, and participants were monetarily compensated for their time ($90).

### Force assessment, exercise execution and fatigue assessment

We assessed the MVC, which is a quantitative measure of muscle strength and represents the maximum amount of force that a person can produce during an isometric exercise^9^. MVC was measured for each participant by using the Biodex 4 Pro equipment (Biodex Medical System, Shirley, New York, USA). This is a sophisticated and reliable equipment that allowed us to assess and control participants’ force during the experiment. To assess the MVC, participants sat down on a chair with the upper part of the body stabilized with belts across the shoulders. They were asked to perform four isometric movements at four different angles (15°, 30°, 45°, 60°). Specifically, participants had to pull on a handle attached to a robotic arm where each movement required 5 seconds of isometric contraction followed by 10 seconds of rest. We set the height of the dynamometer in order to have the center of rotation matching the participant’s elbow position. The arm was fixed with an additional strap in order to help participants perform the exercise correctly. The assessment of the MVC allowed us to set the movement at the 30% of the maximum force for each participant during the experiment in order to reduce the risk of fatigue^10,11^ and standardize the movement for all the participants throughout the procedure

At the end of the MVC assessment, we computed the mean of the peak torque recorded at each position and we calculated the 30% of the MVC. During the force assessment, we recorded the peak velocity of the exercise (degree/second), the average velocity (degree/second), the acceleration and deceleration time (milliseconds). However, during the experimental procedure, velocity (60 deg/sec) and range of motion (i.e. the amplitude of the exercise, 80°) were kept constant for all the participants to standardize the exercise performance. By doing so, we standardized the motor task across participants. During the experiment, participants were asked to perform three extension-flexion movements (i.e. isotonic task) during half of the trials while receiving the thermal stimulation on the dominant forearm. Specifically, the isotonic motor task was performed in 5 trials (15 movements) during the exercise phase, 18 trials (54 movements) during the acquisition phase, and 9 trials (27 movements) during the test phase when placebo and nocebo effects were tested with and without movement task. At the end of each phase, participants rated their level of fatigue on a Borg scale^12^, from 0 (no fatigue) to 10 (extremely severe fatigue).

### Pain stimulation, calibration and assessment

Painful stimulations were delivered by means of the Pathway system (Medoc Advances Medical System, Rimat Yishai, Israel), an equipment delivering heat thermal stimulation starting from 32 °C to the highest deliverable temperature of 50 °C. The heat stimulations were delivered through a 3 × 3 cm probe. Painful stimulations were delivered to the same part of the forearm to avoid the involvement of distinct receptorial areas. We used an elastic bandage to attach the probe gently on the participant’s dominant volar forearm and to ensure it remained stable on the skin area. Importantly, the painful stimulations were delivered on the same forearm executing the movement to maximize the sensory reduction. The pain *calibration* started with the assessment of the warm detection (the level of thermal stimulation perceived as warm but not painful). Participants were then asked to report by pressing the Pathway remoter button, the level of pain that they would perceive as 20 out of 100 on the Visual Analogue Scale (VAS) (i.e. low pain), 50 out of 100 (i.e. medium pain) and 80 out of 100 (i.e. high pain). The identified *highest* level was used as reference to subtract three Celsius degrees to reach the medium level of pain and to reduce from the medium level additional three Celsius degrees to obtain the lowest level of delivered intensity. This procedure allowed as to standardize among participants the levels of temperatures used during the acquisition phase to reinforce expectations (Table 1). The heat painful stimulations were delivered while participants performed the arm extension and flexion exercises during half of the trials and at rest in the other half (control trials). Each heat stimulation lasted 10 seconds and after 2 seconds, participants rated the level of perceived pain (5 sec). Pain expectation and perceived pain was measured using a VAS anchored from 0 (no pain) to 100 (maximum tolerable pain).

### Experimental procedure

**Figure 1:**
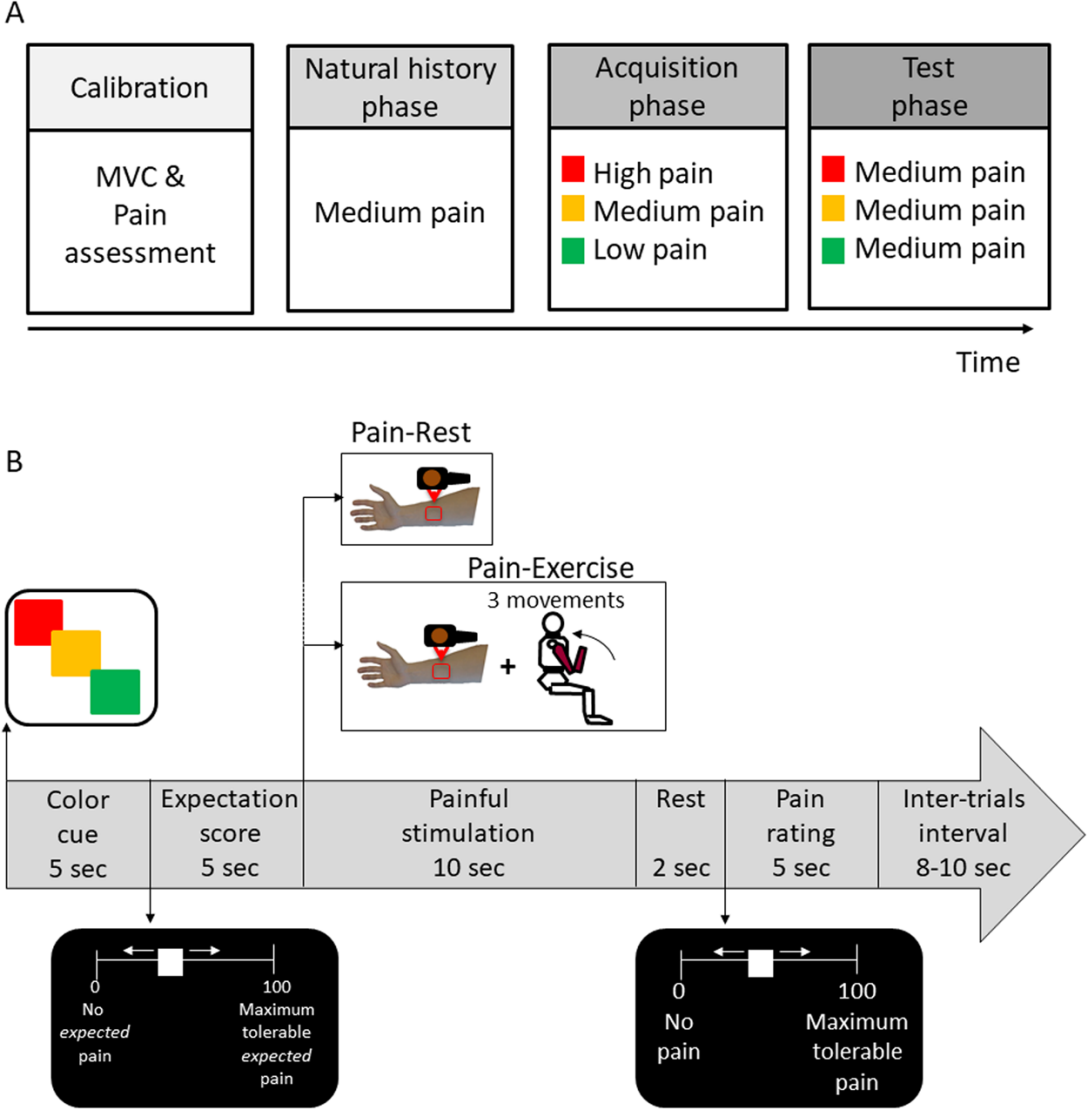
Study design and trial representation. The experiment consisted of one session to calibrate the level of force and pain for each participant, and three subsequent phases. During the *calibration* phase, we assessed the levels of pain necessary to induce a sensation of low, medium, and high pain for each individual. We also assessed the maximum voluntary contraction (MVC) of the dominant arm for each participant. During the *natural history* phase, we delivered a medium level of pain for a total of 10 trials. In half of the trials, participants performed the isotonic movement tailed to their individual MVC. During the *acquisition* phase, the participants learned to associate three visual color cues (red, yellow, green) with three distinct levels of pain (high, medium and low pain intensities) for a total of 12 trials. Participants performed the isotonic movement in half of the trials. During the *test* phase, we set the intensity of the painful stimulations at the same medium level for all the three cues (presented 6 times for each visual color cue) and the isotonic task was introduced in half of the trials. (**A**) During each trial of the acquisition and test phase, after the *color* cue presentation, participants were asked to rate the level of expectation about the upcoming stimulation by means of a 0–100 VAS ranging from no *expected pain* to maximum expected pain. Then, the painful stimulation was delivered for 10 seconds. After two seconds of rest, participants were asked to rate the level of *perceived pain* by means of the 0–100 VAS ranging from 0 = no pain and 100 = maximum tolerable pain. Participants were alerted whether or not to perform the isotonic movement by a sign (e.g. arm performing the movement) displayed on the monitor. An inter-trials interval with variable timing was introduced to avoid habituation (**B**).

Following the calibration of force and pain sensitivity in each participant, the experimental procedure consisted of three phases including: (1) natural history, to test for the effect of the exercise in modulating pain perception prior to any expectancy manipulation; (2) acquisition phase to reinforce expectations by exposing participants to the experience of pain increases and reductions; (3) test phase, to assess the occurrence of nocebo and placebo effects (Fig. 1A).

In all the experimental phases, participants performed the three extension-flexion movements in half of the trials in which they were receiving the painful stimulations. A picture was displayed before the heat stimulation to inform participants whether to perform the exercise or to rest.

An inter-trial-interval of 10 sec was used to prevent habituation (Fig. 1B).

The delivery of event (heat stimulation, visual cues, VAS scales) was managed by pre-programmed scripts using Eprime (Psychology Software Tools, Inc., Sharpsburg, PA, USA; version 2.0). To control for time effects, we counterbalanced the presentation of visual cues during each phase (i.e., Pain-Exercise and Pain-Rest conditions) by assigning participants randomly to one of four sequences. The sequence did not influence the size of placebo and nocebo effects (p = 0.249). In order to maintain constant attention throughout the experimental procedure, participants were asked to count the number of visual color cues presented during the acquisition and test phase trials.

### Natural history phase

**Figure 2:**
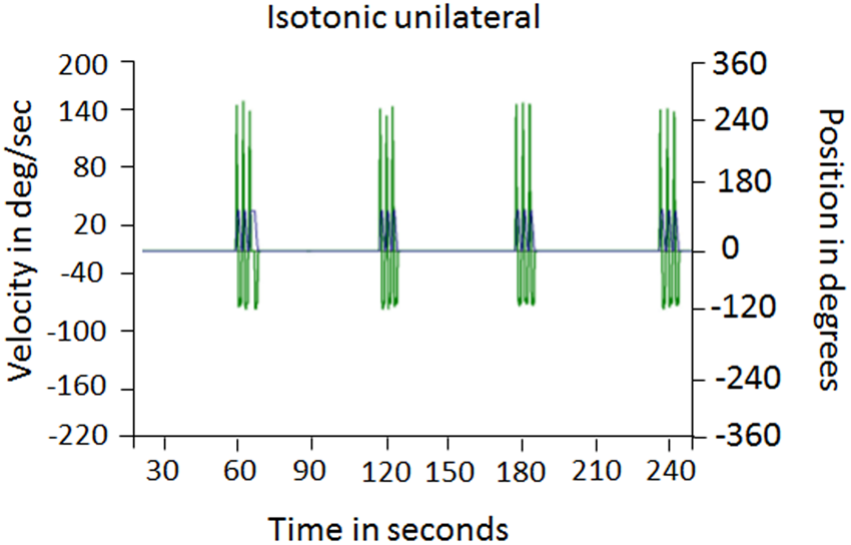
Extension-flexion isotonic movement. Participants performed the extension-flexion isotonic movement while receiving the heat thermal stimulation. The MVC was constantly monitored. The figure shows the Biodex output with the recording of the MVC from a representative participant while performing the movements (Pain-Exercise condition). (**C**) Values are expressed as mean ± sem **p < 0.001 Abbreviations: DEG = degrees, deg/sec = degrees per second.

During the natural history phase, participants received the identified *medium* level of painful stimulation to the volar forearm with 5 trials in which participants completed a motor exercise and 5 trials in which they rested (Fig. 2). Half of the painful stimulations were delivered along with 3 repetitions of elbow extension and flexion exercises set at 30% of the participants’ MVC (Pain-Exercise condition). The remaining painful stimulations were given at rest (Pain-Rest condition). Trials of the Pain-Rest condition were intermixed with Pain-Exercise condition’s trials. This phase tested the hypothesis that the isotonic motor task would have resulted in pain reduction when compared to the same levels of painful stimulations delivered while at rest.

### Acquisition phase

This procedure allowed us to reinforce participants’ expectations based on the type of association between cue and heat intensities (i.e. highest pain with red cue, lowest pain with green cue) as previously performed^1,4^. Participants were told that a red cue would precede the delivery of a moderately high level of painful stimulation, a yellow cue would precede the delivery of a medium level of pain and a green cue would precede a low painful stimulation. In the acquisition phase, participants received the three levels of painful stimulations (high, medium and low).

Each visual cue (red, yellow and green) was presented for 5 sec. Five seconds after the visual cue presentation and before the heat stimulation delivery a VAS scale was displayed to ask participants about their expectation for the upcoming painful experience (“Please rate your expectation about the upcoming pain”). The heat stimulation was therefore delivered and participants were asked to rate on the 0–100 VAS scale the perceived painful sensation (“Please rate your perceived pain”). The acquisition phase consisted of a total of 36 trials divided into two sessions of 18 trials each, with half of them combined with the extension and flexion task.

### Test Phase

During the *test* phase, participants were shown each visual cue (red, yellow and green) 6 times, for a total amount of 18 painful stimulations with half of them combined with the extension and flexion task. All thermal stimulations were delivered at a medium intensity regardless of the color cue presented. Nine painful trials were intermixed with three elbow extension-flexion exercises set at 30% of the MVC. For each trial, after the visual cue presentation (5 sec), participants rated their expectation (5 sec) on the 0–100 VAS. After each painful stimulus was delivered, participants rated their perceived pain.

### Data Analysis

Based on previous studies that have applied a similar paradigm^11,13^, we expect an effect size of d = 0.32. Assuming an anticipated effect size of d = 0.32 and a p-value (or type I error rate) equal to 0.05 to claim statistical significance the required sample size is n = 34 participants. Power analyses were conducted with G*Power 3^14^. Given that there were no published data regarding the modulation of exercise-induced hypoalgesia and we included both men and women, the enrollment was n = 50 participants.

In the natural history phase, VAS pain reports were analyzed using repeated measures (rm) ANOVA with Condition (Pain-Rest vs. Pain-Exercise) and Trials as within-subject factors.

During the acquisition and test phases, expectation and pain VAS scores were collected on a trial-by-trial basis. VAS scores for expectations and perceived pain were analyzed separately in the acquisition and test phase using rmANOVA with Condition (Pain-Rest vs. Pain-Exercise), Color cue (red, yellow, green) and Trials as within-subject factors. Moreover, an additional analysis was conducted in order to compare any significant Pain-Rest vs. Pain-Exercise effect across phases by means of rmANOVA with Condition (Pain-Rest vs. Pain-Exercise), Color cue (red, yellow, green) and Phases (2 block acquisition vs. test phase) as within-subject factors. Post-hoc comparisons and paired t-tests with Bonferroni adjustment for multiple comparisons were conducted where necessary. Placebo effects were operationally defined as the difference between Green-Yellow associated trials and nocebo effects were operationally defined as Red-Yellow associated trials. The exercise-induced hypoalgesia was defined as the difference in pain ratings between the Pain-Exercise and Pain-Rest conditions. The Pain-Exercise and Pain-Rest difference was used to compare the level of pain changes induced by placebo and nocebo manipulations by means of Univariate ANOVA.

Spearman coefficient (1-tailed, given the unidirectional hypotheses) was used to correlate placebo and nocebo effects with expectations, pain sensitivity (i.e. level of intensity used to elicit medium pain) and fatigue levels. Sex effects were tested by independent samples t-test including the comparison between the follicular versus the luteal phase (women). The strength of two correlations was compared using the Fisher r-t-z transformation.

All the analyses were performed using the Statistical Package for the Social Sciences (SPSS) software package (SPSS Inc, Chicago, Illinois, USA, vers. 21). The level of significance for all analyses was set at p ≤ 0.05.

## Results

### Natural history phase

**Figure 3:**
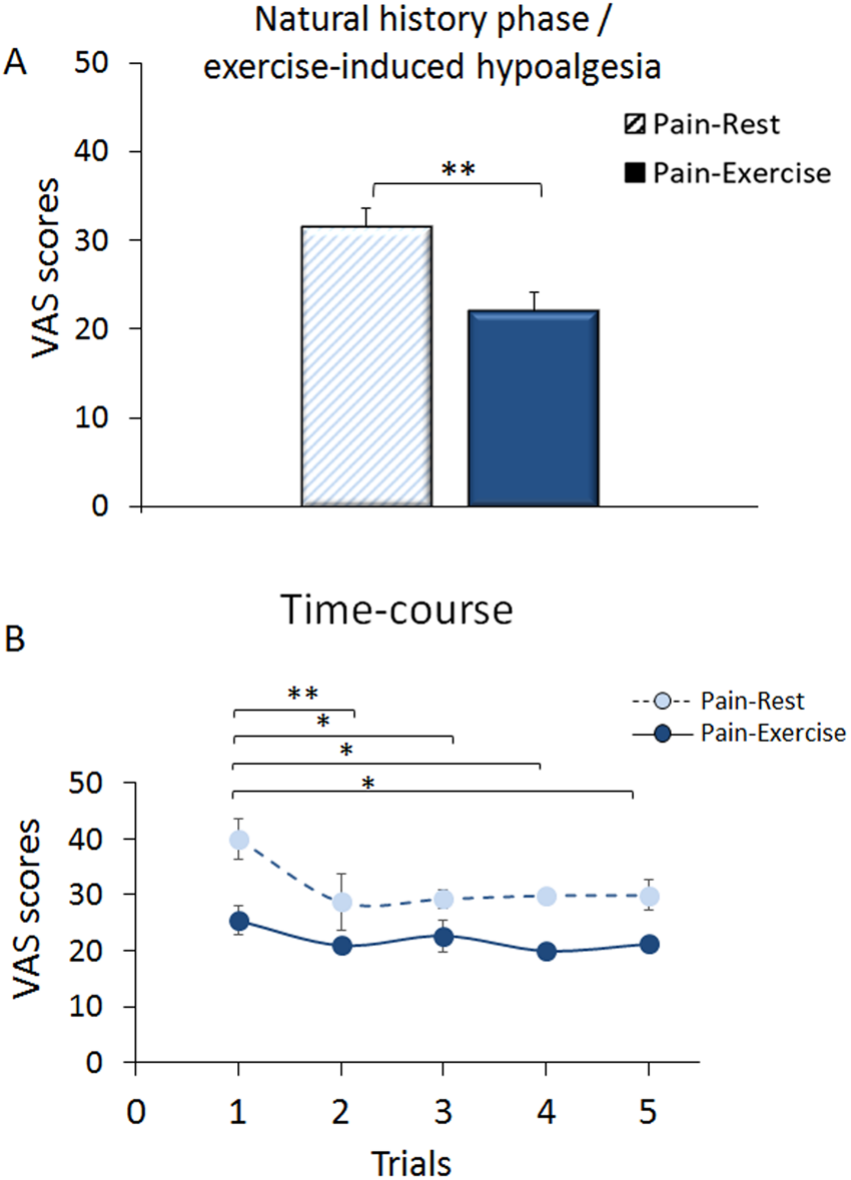
Natural history phase/exercise-induced hypoalgesia. During the exercise alone phase, the same medium level of pain intensity was given without displaying any color cues and performing any pain manipulations to reinforce expectations. Participants performed the isotonic movement in half of the trials. Participants reported as less painful the heat thermal stimulations delivered while performing the exercise (Pain-Exercise condition, full bar) compared to the rest condition (Pain-Rest condition, striped bar). (**A**) The hypoalgesic effect changed over time with the first set of trials significantly different than Trials 2, 3, 4 and 5, respectively (**B**).

The effect of exercise on pain was explored before any placebo or nocebo manipulation and all the stimulations were delivered at a medium level of pain as derived from an initial calibration procedure. In the natural history phase, participants reported that the same medium heat stimulations were perceived as less painful in the Pain-Exercise (mean ± sem, 22.05 ± 2.11) than the Pain-Rest condition (31.54 ± 2.05) (Condition, F_(1,45)_ = 9.489, p = 0.004), suggesting that performing an isotonic exercise during the delivery of a heat painful stimulation induced per se a hypoalgesic effect (Fig. 3A). The factor Trials was significant (Trials, F_(1,45)_ = 14.968, p < 0.001, Fig. 3B) with a significant Condition*Trials interaction (F_(1,54)_ = 9.727; p = 0.003). Trials 1 were significantly different than Trials 2 (p < 0.001), 3 (p = 0.027), 4 (p = 0.011) and 5 (p = 0.019) indicating that the effect reduced over time (Fig. 3B).

### Acquisition phase

**Figure 4:**
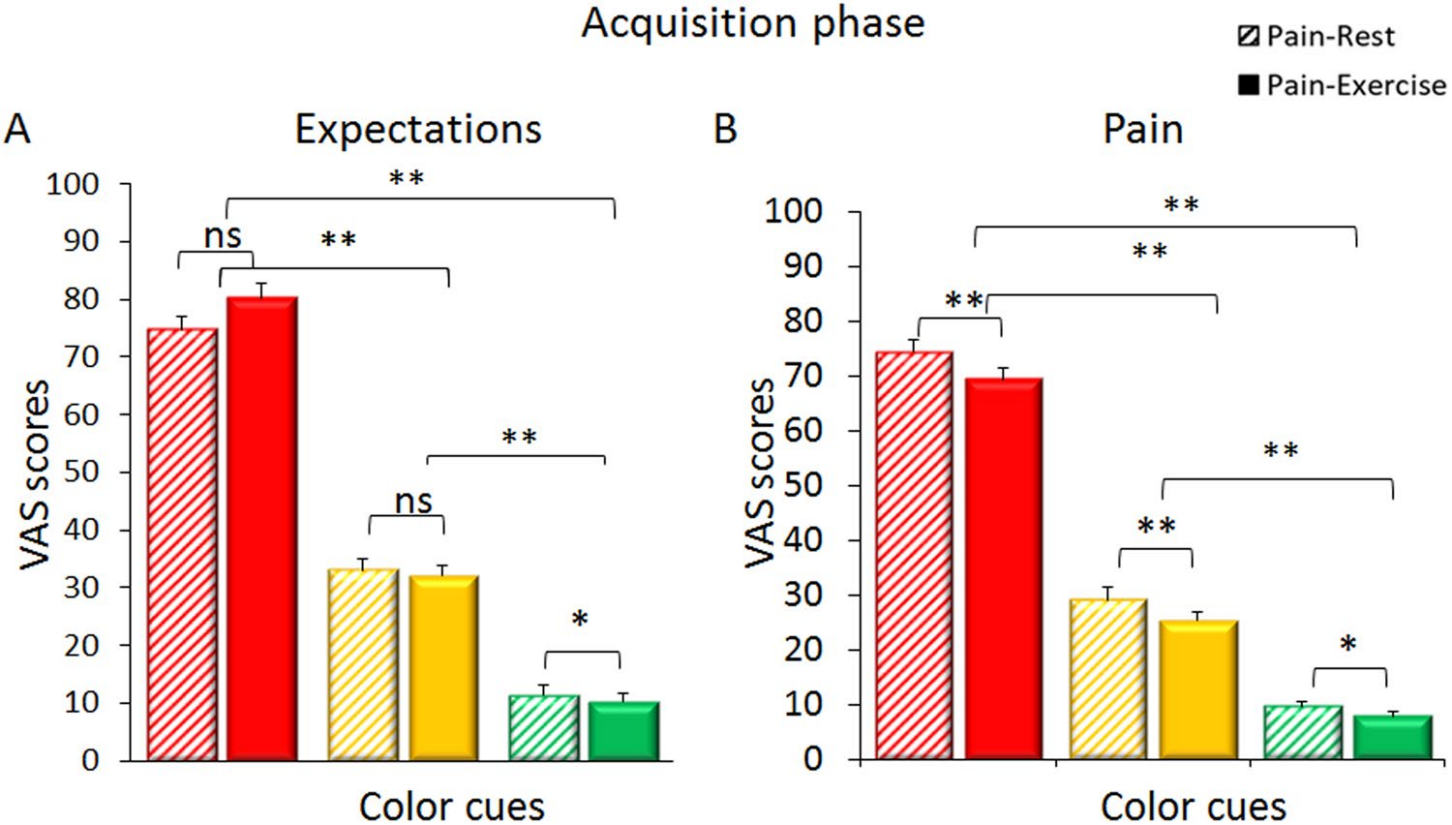
Expectation and pain scores in the acquisition phase. During the *acquisition* phase, expectations scores reflected the anticipation of three distinct expected levels of pain in accordance with the color cue (the colors of the bars represent the colors of the cues) and the verbal instructions. (**A**) Pain ratings given at rest (Pain-Rest condition, striped-color bars) and during the extension-flexion motor task (Pain-Exercise condition, full-color bars) confirmed that participants had learned to distinguish the three intensities of pain (i.e., high after the red cue, medium after the yellow cue and low after the green cue). (**B**) The thermal stimulations received in the Pain-Exercise condition were perceived as less painful than the stimulations received in the Pain-Rest condition further confirming the exercise-induced hypoalgesic effect.

During this phase, expectation and pain VAS scores were collected for each trial. The analysis of *expectation* scores revealed a significance for the factor Color cue (F_(2,90)_ = 560.412, p < 0.001). This indicates that participants learned to anticipate that the stimulations preceded by the red cue were more painful (VAS score: 77.50 ± 16.36) than those preceded by the yellow (control) cue (32.56 ± 11.60; red vs yellow cue associations, p < 0.001) and the stimulations preceded by the green cue were less painful (10.78 ± 6.40) than those preceded by the yellow cue (green vs yellow, p < 0.001) (Fig. 4A). The factor Trials was not significant (Trials, F_(5,225)_ = 2.158, p = 0.06).

Similarly, *pain* scores showed a main effect of the Color cue (F_(2,90)_ = 449.588, p < 0.001). Painful stimulations were reported as more painful when associated with the red (71.87 ± 16.63) than the yellow cue (27.18 ± 9.82, red vs yellow, p < 0.001) and less painful when associated with the green (8.60 ± 5.87) than the yellow cue (green vs yellow cue associations, p < 0.001) (Fig. 4B). There was a main effect of the factor Trials (Trials, F_(5,225)_ = 3.154, p = 0.009) with a significant Condition*Color cue* Trials interaction (F_(10,450)_ = 4.597; p < 0.001).

Painful stimulations at Rest were more painful than those accompanied by the exercise (Red: Pain-Rest vs Pain-Exercise: 74.42 ± 2.30 and 69.31 ± 2.78; t_(45)_ = 3.610; p < 0.001; Yellow: Pain-Rest vs Pain-Exercise: 29.16 ± 1.53 and 25.24 ± 1.55; t_(45)_ = 3.705; p < 0.001; Green: Pain-Rest vs Pain-Exercise: 9.5 ± 1 and 7.68 ± 0.87; t_(45)_ = 2.515; p = 0.016) (Fig. 4B), further confirming that the exercise induced a hypoalgesic effect, participants learned to discriminate the three level of pain intensity and their expectations were tuned consistently with the upcoming heat stimulations.

In accordance with our hypothesis, the heat painful stimulations delivered during the Pain-Exercise condition (34.08 ± 1.28) were perceived as less painful than those delivered in the Pain-Rest condition (37.69 ± 1.20) (Condition, F_(1,45)_ = 22.657, p < 0.001).

### Test phase

**Figure 5:**
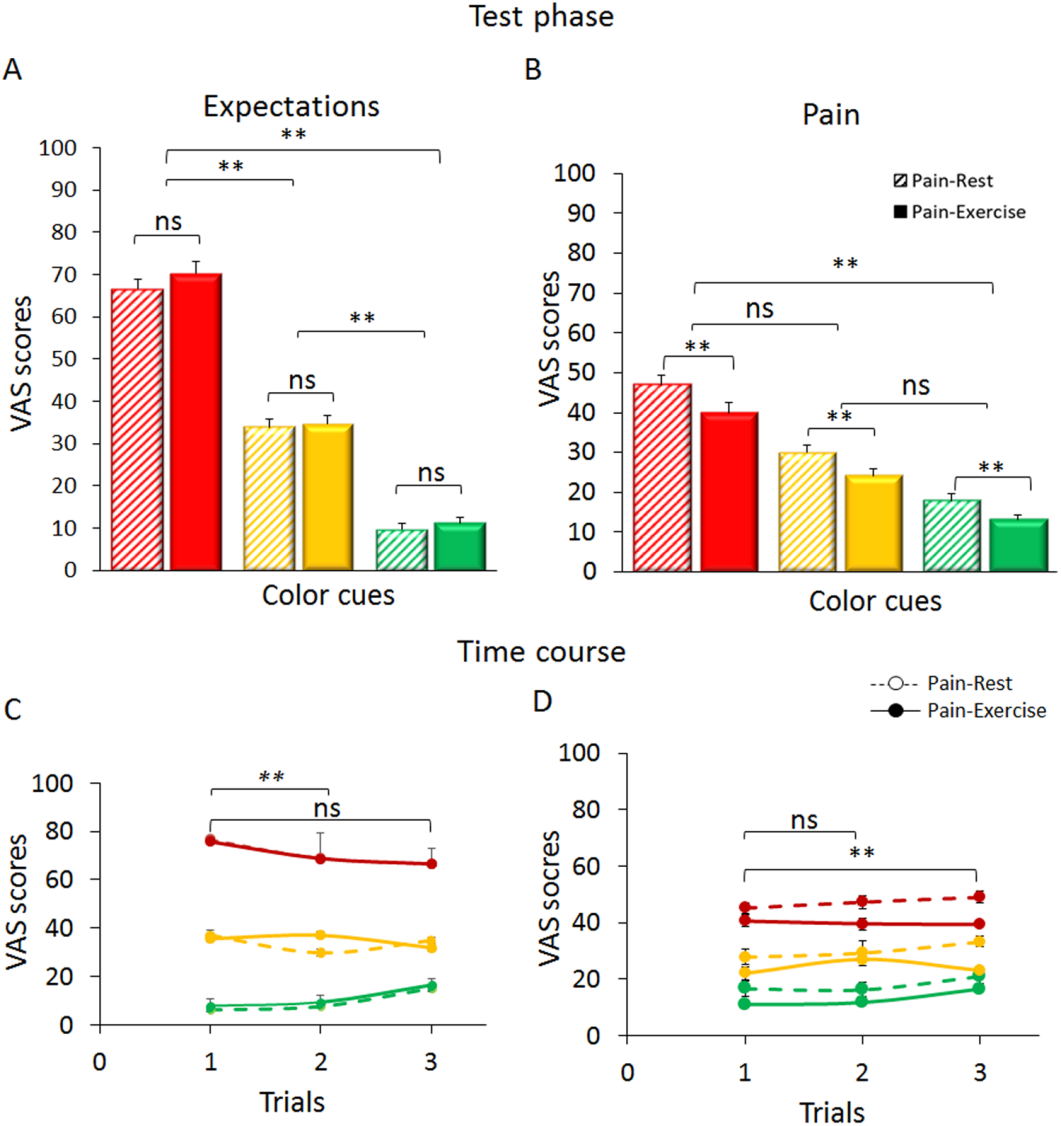
Expectation and pain scores in the test phase. During the *test* phase (when all painful stimulations were set at the medium level of intensity), participants reported three different levels of expected pain (**A**) and three levels of perceived pain (Pain-Rest condition, striped-color bars) at rest and during the extension-flexion exercises (Pain-Exercise condition, full-color bars). (**B**) The time-courses of expectations (**C**) and pain reports (**D**) indicated relatively stable trends over the test sessions. Although, participants perceived as less painful the stimulations given in conjunction with the motor task, the delta (Green-Yellow at Rest vs Green-Yellow during the exercise and Red-Yellow at Rest vs Red-Yellow during the exercise, respectively) were not affected indicating no additive effects of the isotonic movement on placebo and nocebo effects. All data are expressed as mean ± sem. **p < 0.001; *p < 0.05; *ns* = not significant. Abbreviation: Exerc = Exercise.

In the test phase, when we set surreptitiously all the painful stimulations at the medium level of intensity, *expectation* VAS scores showed that despite the violation between sensory stimulations and cues, participants continued expecting low, medium and high upcoming pain (Color cue, F_(2,90)_ = 436.825, p < 0.001). Post-hoc comparisons showed that the stimulations preceded by the red cue were expected as more painful (70.49 ± 16.01) than those preceded by the yellow cue (34.34 ± 11.50, red vs yellow, p < 0.001) and those preceded by the green cue (10.40 ± 6.42) to be less painful than those preceded by the yellow cue (green vs yellow cue associations, p < 0.001) (Fig. 5A). The factor Trials was significant (Trials, F_(2,90)_ = 9.567, p < 0.001) with a significant Condition*Color cue* Trials interaction (F_(4,180)_ = 4.385; p = 0.002). Post-hoc comparisons indicated that the first set of trials (red, yellow, green rest and motor conditions, respectively) differed from trials 2 (p < 0.001) but not from trials 3 (p = 0.239) (Fig. 5C).

Remarkably, despite the intensity of stimulation being the same for all cue-pain associations, the VAS *pain* ratings followed the same pattern as expectations (Color cue, F_(2,90)_ = 109.843, p < 0.001). Overall, participants reported that they perceived as more painful stimulations given after the red cue (43.44 ± 16.41) as compared to the yellow cue (26.99 ± 11.55, red vs yellow, p < 0.001), and they perceived as less painful stimulation associated with the green anticipatory cue (15.44 ± 9.48) than after the yellow cue (green vs yellow, p < 0.001) (Fig. 5B), indicating the presence of significant nocebo and placebo effects.

**Figure 6:**
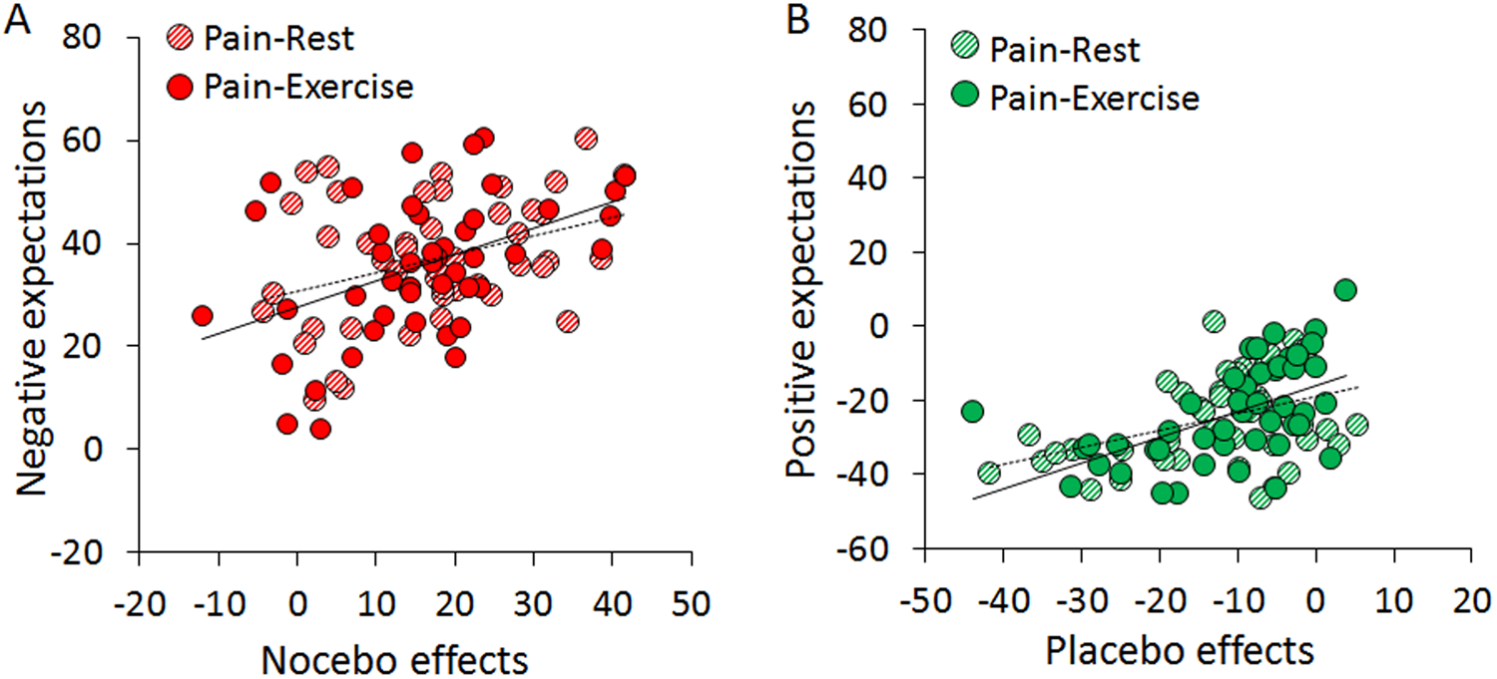
Expectations, placebo and nocebo effects. VAS pain ratings for nocebo (**A**) and placebo (**B**) effects were significantly correlated with expectation scores in the test phase for both the Pain-Rest (striped circles) and in Pain-Exercise (full circles) conditions.

Moreover, we tested for the effects of exercise in conjunction with placebo and nocebo. We observed that the stimulations delivered during the Pain-Exercise condition were overall perceived differently than those delivered in the Pain-Rest condition (Condition, F_(1,45)_ = 33.703, p < 0.001), however the Color cue*Condition interaction was not significant (F_(2,90)_ = 1.167, p = 0.316), Fig. 5D. The factor Trials was significant (Trials, F_(2,90)_ = 7.383, p = 0.001) but with no significant Condition*Color cue*Trials interaction (F_(4,180)_ = 1.811; p = 0.129). Post-hoc comparisons showed that the set of the first trials did not differ from the second one (p = 0.476) but differed from trials 3 (p < 0.001) (Fig. 5D). Expectation scores were associated with the placebo and nocebo effects, both during exercise and at rest (Pain-Rest: nocebo: r = 0.290, p = 0.050; Pain-Exercise, nocebo: r = 0.444, p = 0.002; placebo: r = 0.368, p = 0.012; placebo: r = 0.592, p < 0.001] (Fig. 6A,B). These correlations appear to be equal in significance as revealed by the Fisher z values (z = −0.83; p = 0.203 for nocebo and z = −1.37; p = 0.085 for placebo conditions), suggesting that exercise and cue-driven placebo and nocebo effects are likely to work independently.

### Comparison across phases

The hypoalgesic contribution of exercise was larger in the test phase than in the acquisition phase (Phase, F_(1,45)_ = 7.148, p = 0.010), independently of the color cue (Color cue*Phase interaction F_(2,90)_ = 0.157, p = 0.855), suggesting that exercise-induced hypoalgesia could be enhanced throughout training.

Finally, we compared the pain changes induced by exercise, placebo and nocebo manipulation alone and found that the three procedures differed significantly (F_(2,135)_ = 104.291; p < 0.001). Post-hoc comparisons indicated that the hypoalgesic effect induced by the exercise (natural history phase) and by the placebo manipulation (test phase) was not different (p = 0.771), indicating no additive effect. By contrast, the nocebo manipulation was significantly different in magnitude than both placebo (p < 0.001) and exercise alone (p < 0.001).

### Effects of perceived fatigue, level of pain used and sex

At the end of each phase, participants rated their level of fatigue on a Borg scale, from 0 (not at all) to 10 (extremely severe). To rule out any influence of fatigue on the placebo and nocebo effects obtained in the test phase, correlational analyses (Spearman) were performed between the scores at the Borg scale and the placebo and nocebo responses. No significant correlation was found neither in the Pain-Exercise condition (placebo: r = 0.175, p = 0.246; nocebo: r = 0.156, p = 0.299) nor in the Pain-Rest condition (placebo: r = 0.038, p = 0.803; nocebo: r = 0.128, p = 0.395).

Finally, we explored whether individual pain as reflected by the level of intensity used to elicit medium pain, affected the magnitude of placebo analgesia and exercise-induced analgesia. We found no influences of pain levels on exercise-induced hypoalgesia (r = −0.056, p = 0.357), placebo induced analgesia without the isotonic task (r = −0.085, p = 0.288), and placebo induced analgesia with the isotonic task (r = −0.058, p = 0.351). The individual level of pain correlated inversely with nocebo effects observed at rest (r = −0.280; p = 0.03) but not during the execution of the isotonic task (r = −0.031; p = 0.419). This inverse correlation may indicate that participants who were more tolerant, received higher heat stimulations and exhibited lower nocebo effects likely because of less anxiety/arousal towards painful stimulation.

Additionally, we determined the influence of sex and female menstrual phases. We found no difference between men and women with regards to placebo (t_(44)_ = 0.755, p = 0.454), nocebo (t_(44)_ = −0.845, p = 0.403), and exercise-induced hypoalgesia (t_(44)_ = −0.723, p = 0.473). Moreover, we checked for any difference due to the menstrual cycle phases in women. Out of 24 women participants, 10 completed the study during the follicular phase and 11 during the luteal phase. Three of them were excluded from this sub-analysis because of the menopause period and absence of menstruation due to birth control methods. We found that placebo (t_(19)_ = 1.786, p = 0.090), nocebo (t_(19)_ = −0.127, p = 0.900) and expectations (positive: t_(19)_ = 0.064, p = 0.949; negative: t_(19)_ = −0.713, p = 0.484) were not influenced by the follicular cycle. By contrast, women who participated in the study during the luteal phase displayed stronger exercise-induced hypoalgesic effect as compared to those in the follicular phase (test phase, t_(19)_ = −2.802, p = 0.011). Future research is needed to understand how gonadal hormones may influence exercise-induced hypoalgesia.

## Discussion

The aim of this study was to investigate the interplay between exercise, placebo and nocebo effects on pain modulation. We demonstrated in a within-subject study design that both exercise and placebo effects reduce heat painful sensations. The pain reductions induced by placebo effects via reinforced positive expectations and exercise were equal in magnitude suggesting that placebo- and exercise-induced hypoalgesia may represent two important distinct modalities of pain inhibition. Importantly, nocebo effects were nevertheless present even during exercise, as revealed by the higher pain ratings following the red cue compared to the green and yellow cues during the motor task.

Exercise, placebo (and nocebo) are likely to work throughout distinct pain modulatory systems. Further mechanistic research that combines exercise and expectation manipulations could provide potential new strategies for optimizing nonpharmacological pain interventions.

Recent studies have demonstrated that motor performance in athletes, non-athletes and patients with motor deficits can be bi-directionally modulated by expectations, placebo and nocebo effects^10,15–21^. For example, Benedetti and colleagues (2007) showed that pharmacological conditioning with opioids improves pain endurance and motor performance in study participants who underwent a submaximal effort tourniquet technique^19^. Maganaris and colleagues (2000) enrolled 21 athletes to study effects of deceptive administration of anabolic steroids on the maximum force production during bench press, dead lift, and squat exercises. Results showed an improvement and a maintenance of the performance in participants who believed they ingested the steroids^22^. Further studies showed that placebo procedures can improve different parameters of motor performance, such as force^10^, resistance to fatigue^23^, and speed^24^. Beedie and colleagues (2007) investigated the role of positive versus negative beliefs on repeated 30m-sprint performances^24^. Participants were informed they had received an ergogenic aid and they received information about either the positive or the negative impact of the treatment on the performance. Meanwhile, all the participants received an inert substance, the group informed about the improvement of both endurance and performance showed an enhancement of the speed. Conversely, those who were informed about the negative effect on the performance, recorded a worsening of the speed^24^. Similarly, a decrease of muscle work was found in non-athletes during a leg extension exercise after the application of a sham electrical device that the participants thought to have negative effects^25^.

Herein, we expanded placebo and nocebo research in the contexts of pain and motor performance using a within-subjects design combined approach. Pain and motor performance have primarily been investigated separately and tested with different protocols. Deliberately, we created a combined experimental paradigm in which painful heat stimulations were delivered during the execution of isotonic task. Both painful stimuli and movements were tailored to the individual pain sensitivity (i.e. individual level of medium heat pain) and strength (i.e., 30% of the maximal strength) respectively. Consistent with the literature on exercise-induced analgesia^26–29^, we found that the isotonic task reduced pain perception when heat stimulation was delivered at the same time as the movement. We compared in the same study participants the reduction of experimental pain induced by exercise per se, placebo effects (via reinforced positive expectations) with and without the execution of the isotonic task. Importantly, the exercise and placebo with motor task equally reduced the pain experience. The nocebo hyperalgesic effect remained significantly high irrespectively of the condition (Rest vs Exercise). This may suggest that exercise does not abolish the nocebo hyperalgesic effect, likely because of the strength of negative expectations in inducing nocebo effects^30,31^.

These effects might be interpreted in at least two distinct ways. First, sensory reduction that has been proposed as a characteristic of voluntary movement^32–35^ may have shaped the integration of sensorial and motor inputs generating a reduction of perceived intensity of pain. Within this frame of reference, expectations about the pain may have interfered with the predictions of pain and the sensory feedback. For example, it has been suggested that whenever we perform an action, copies of the motor command are dispatched as corollary discharges to sensory structures that predict the sensory consequences of the action^36–38^. Internal predictions about the movement execution and upcoming painful stimuli were likely integrated and finely shaped by expectations.

Second, attention may have played a role. Some may argue that exercise execution *per se* capitalized participants’ attention towards the movement, thus distracting them from the painful stimuli and consequently making them feel less pain. Although we did not explicitly measure the level of attention, we are inclined to think that this is not the case based on the fact that the modulation of pain during the execution of the isotonic movement was constantly present in the acquisition and test phases as well as for all the cues (red, yellow, green). Interestingly, expectation scores were significantly correlated with both placebo and nocebo effects during movement execution expanding our previous results^1^. The individual level of used pain correlated inversely with nocebo effects. This inverse correlation may indicate that participants who were more tolerant, received higher heat stimulations and exhibited lower nocebo effects likely because of less anxiety/arousal towards pain^1^. Another important aspect is that those subjects who displayed placebo or nocebo responses at rest, continued to be responders in the movement condition adding to the reproducibility of placebo effects^39^. While exercise and placebo-induced hypoalgesia appear to be two distinct sides of the pain modulation phenomenon, our innovative result opens interesting perspectives in the placebo and nocebo research, with potential implications for sport, rehabilitation and pain management.

Some pitfalls and limitations remain to be discussed. Due to equipment limitations, we did not include a control condition in which the contralateral arm was stimulated at rest. In addition, we did not ask participants whether or not they engaged in regular physical activity. However, we recruited from a student population and the participants were screened for healthy conditions^40^. Furthermore, to better and deeply understand the role of attention, future studies may investigate the extent to which attention (i.e. addition of a working memory task) affects the perception of pain during a well-controlled movement execution, thus identifying an additional factor that helps to reduce the experience of pain. This paradigm is suitable for future mechanistic studies in which exercise-induced hypoalgesia, expectations, and violation of expected outcomes can be investigated with neurophysiological approaches (i.e. TMS). For example, Fiorio and colleagues (2014), by applying transcranial magnetic stimulation over the primary motor cortex, found that a placebo-induced modulation of force was paralleled by changes in the excitability of the corticospinal system as shown by increased amplitude of the motor evoked potentials and decreased duration of the cortical silent period, hinting of a cognitive enhancement of corticospinal excitability^10^. Nocebo manipulations are also associated to corticospinal changes^11^.

In sum, our study was innovative in nature because it demonstrates that two modalities of descending pain systems can be simultaneously activated to gain hypoalgesia opening up new research avenues with important mechanistic approaches and potential clinical applications for sport, rehabilitation and pain medicine.

## Conclusion

Herein, we demonstrated that pain sensations can be reduced by both the execution of an isotonic exercise and the reinforcement of participants’ expectations, thus highlighting two important modalities of pain inhibition. Translating these findings in the real-world setting is of utmost importance for pain management in clinical settings, in order to optimize not only rehabilitation programs, but also therapeutic strategies and treatment outcomes.
